# STAT3 Controls the Long-Term Survival and Phenotype of Repair Schwann Cells during Nerve Regeneration

**DOI:** 10.1523/JNEUROSCI.3481-16.2017

**Published:** 2017-04-19

**Authors:** Cristina Benito, Catherine M. Davis, Jose A. Gomez-Sanchez, Mark Turmaine, Dies Meijer, Valeria Poli, Rhona Mirsky, Kristjan R. Jessen

**Affiliations:** ^1^Department of Cell and Developmental Biology, University College London, London WC1E 6BT, United Kingdom,; ^2^Centre for Neuroregeneration, Edinburgh EH16 4SB, United Kingdom, and; ^3^Department of Molecular Biotechnology and Health Sciences, University of Turin, 10126 Torino, Italy

**Keywords:** denervation, injury, nerve, regeneration, repair, Schwann

## Abstract

After nerve injury, Schwann cells convert to a phenotype specialized to promote repair. But during the slow process of axonal regrowth, these repair Schwann cells gradually lose their regeneration-supportive features and eventually die. Although this is a key reason for the frequent regeneration failures in humans, the transcriptional mechanisms that control long-term survival and phenotype of repair cells have not been studied, and the molecular signaling underlying their decline is obscure. We show, in mice, that Schwann cell STAT3 has a dual role. It supports the long-term survival of repair Schwann cells and is required for the maintenance of repair Schwann cell properties. In contrast, STAT3 is less important for the initial generation of repair Schwann cells after injury. In repair Schwann cells, we find that Schwann cell STAT3 activation by Tyr705 phosphorylation is sustained during long-term denervation. STAT3 is required for maintaining autocrine Schwann cell survival signaling, and inactivation of Schwann cell STAT3 results in a striking loss of repair cells from chronically denervated distal stumps. STAT3 inactivation also results in abnormal morphology of repair cells and regeneration tracks, and failure to sustain expression of repair cell markers, including Shh, GDNF, and BDNF. Because Schwann cell development proceeds normally without STAT3, the function of this factor appears restricted to Schwann cells after injury. This identification of transcriptional mechanisms that support long-term survival and differentiation of repair cells will help identify, and eventually correct, the failures that lead to the deterioration of this important cell population.

**SIGNIFICANCE STATEMENT** Although injured peripheral nerves contain repair Schwann cells that provide signals and spatial clues for promoting regeneration, the clinical outcome after nerve damage is frequently poor. A key reason for this is that, during the slow growth of axons through the proximal parts of injured nerves repair, Schwann cells gradually lose regeneration-supporting features and eventually die. Identification of signals that sustain repair cells is therefore an important goal. We have found that in mice the transcription factor STAT3 protects these cells from death and contributes to maintaining the molecular and morphological repair phenotype that promotes axonal regeneration. Defining the molecular mechanisms that maintain repair Schwann cells is an essential step toward developing therapeutic strategies that improve nerve regeneration and functional recovery.

## Introduction

The regeneration of damaged nerves depends on the presence of living Schwann cells in the nerve distal to injury. These cells are derived from myelin and Remak cells but have adopted a phenotype that is specialized for supporting nerve repair (Arthur-Farraj et al., 2012; Jessen et al., 2015; Jessen and Mirsky, 2016). Repair Schwann cells form regeneration tracks (bands of Büngner) that guide axons to their targets, break down myelin both directly by myelin autophagy and indirectly by activation of the innate immune response and recruitment of macrophages, and express trophic factors that support survival of injured neurons and axon growth (Chen et al., 2007; Arthur-Farraj et al., 2012; Fontana et al., 2012; Glenn and Talbot, 2013; Brosius Lutz and Barres, 2014; Gomez-Sanchez et al., 2015). These cells differ in molecular expression, morphology, function, and transcriptional controls from immature Schwann cells in developing nerves (Jessen and Mirsky, 2016).

Although peripheral nerves respond in this strikingly adaptive fashion to damage, the clinical outcome after nerve injury in larger animals, including humans, is frequently poor (Allan, 2000; Lundborg, 2000; Höke, 2006). One of the main reasons is that, during the slow growth of axons through the more proximal parts of injured nerves, the more distal nerve, which is without axonal contact for extended periods, gradually loses the capacity to support regeneration (Sulaiman and Gordon, 2009, 2013; Scheib and Höke, 2013). Two factors are thought to contribute to this deterioration. One is the gradual death of chronically denervated Schwann cells (Weinberg and Spencer, 1978; Siironen et al., 1994; Li et al., 1998; Jonsson et al., 2013). The other is the reduction in expression of growth-supportive factors including GDNF and BDNF by the surviving cells (You et al., 1997; Höke et al., 2002; Michalski et al., 2008; Eggers et al., 2010). This fading of the repair Schwann cell phenotype and the accompanying loss of regenerative support provided by the distal nerve stump have been carefully analyzed in rodent models of chronic denervation (Sulaiman and Gordon, 2009). Identification of the molecular mechanisms that sustain the differentiation state of repair cells and support their continual survival is clearly a significant goal.

We have previously identified activation of the transcription factor c-Jun in Schwann cells as an important regulator of the reprogramming of myelin and Remak cells into repair Schwann cells (Arthur-Farraj et al., 2012; Jessen and Mirsky, 2016). But transcriptional mechanisms that control long-term maintenance of these cells have not been studied. Here we show that the STAT3 is involved in supporting the survival of chronically denervated repair cells, and also in maintaining their characteristic gene expression and morphology.

STAT3 is typically activated by phosphorylation of conserved tyrosine 705 residue in the C-terminal domain, resulting in dimerization and translocation from the cytoplasm to the nucleus (Aaronson and Horvath, 2002). Signaling is generally mediated via the gp130 receptor complex and Janus kinases (JAKs). STAT3 can also be phosphorylated on serine 727, which in most often serves to augment signaling initiated by tyrosine 705 phosphorylation (Decker and Kovarik, 2000).

STAT3 has previously been implicated in the injury response of CNS glial cells because it is important for the formation of the astrocyte glial scar (Wanner et al., 2013). In Schwann cells, STAT3 is also known to be phosphorylated after injury (Sheu et al., 2000; Lee et al., 2009a, b), but the functional role of STAT3 activation in Schwann cells has not been investigated. In the present work, we have addressed this issue during nerve development, regeneration, and after long-term injury. While we do not find major function for STAT3 in Schwann cell development or myelination, we identify an important role in the maintenance of chronically denervated repair Schwann cells. STAT3 is therefore the second transcription factor, in addition to c-Jun, with a selective function in Schwann cells of injured adult nerves.

## Materials and Methods

### 

#### 

##### Animals.

Animal experiments conformed to United Kingdom Home Office guidelines under the supervision of University College London Biological Services. Sprague Dawley rat pups of either sex were obtained from University College London Biological Services. Mice of either sex with specific deletion of the STAT3 gene in Schwann cells were obtained by crossing STAT3^f/f^ mice (Alonzi et al., 2001) with P_0_-Cre mice (Feltri et al., 2002; D'Antonio et al., 2006), or with Dhh-Cre mice (Jaegle et al., 2003) (for experiments in Figs. 3*D*, 4*C*, 6*A–E*). The resulting P_0_-Cre^+^/STAT3^f/wt^ mice were crossed back to STAT3^f/f^ mice to obtain P_0_-Cre^+^/STAT3f/f mice, referred to as STAT3 cKO mice in which STAT3 is deleted from Schwann cells. P_0_-Cre^−^/STAT3^f/f^ littermates, referred to as WT, were controls.

##### Genotyping.

DNA for genotyping was was extracted from ear or tail samples using the Hot Sodium Hydroxide and Tris method (HotSHot) as in Gomez-Sanchez et al. (2015). For primers, see Table 1.

**Table 1.** Primers for qPCR and genotyping*^a^*

Gene		Accession	Sense sequence	Antisense sequence
Gapdh	Glyceraldehyde-3-phosphate dehydrogenase	NM_001289726.1	AGGTCGGTGTGAACGGATTTG	TGTAGACCATGTAGTTGAGGTCA
Canx	Calnexin	NM_007597.3	CAACAGGGGAGGTTTATTTTGCT	TCCCACTTTCCATCATATTTGGC
c-Jun	c-Jun	NM_010591.2	CCTTCTACGACGATGCCCTC	GGTTCAAGGTCATGCTCTGTTT
Olig1	Oligodendrocyte transcription factor 1	NM_016968.4	CCGCCCCAGATGTACTATGC	AACCCACCAGCTCATACAGC
Shh	Sonic hedgehog	NM_009170.3	AAAGCTGACCCCTTTAGCCTA	TTCGGAGTTTCTTGTGATCTTCC
GDNF	Glial cell-derived neurotrophic factor	NM_010275.3	GATTCGGGCCACTTGGAGTT	GACAGCCACGACATCCCATA
BDNF	Brain-derived neurotrophic factor	NM_007540.4	TCATACTTCGGTTGCATGAAGG	AGACCTCTCGAACCTGCCC

*^a^*Primers used for genotyping STAT3f/f mice are 5′-CAC CAA CAC ATG CTA TTT GTA GG-3′ and 5′-CCT GTC TCT GAC AGG CCA TC-3′ (210 bp band for WT allele and 370 bp band flox allele). Primers for the STAT3 deleted flox allele (310 bp band) are 5′-CAC CAA CAC ATG CTA TTT GTA GG-3′ and 5′-GCA GCA GAA TAC TCT ACA GCT C-3′. Primers for the P0-Cre transgene are 5′-GCTGGCCCAAATGTTGCTGG-3′ and 5′-CCACCACCTCTCCATTGCAC-3′ (480 bp band; Feltri et al., 2002).

##### Antibodies.

P-STAT3-Ser727 and P-STAT3-Tyr705 antibodies, both from Cell Signaling Technology, were used at 1:50 for immunohistochemistry and 1:2000 for Western blotting. Other antibodies for Western blotting were Cyclin D1 (1:200; Santa Cruz Biotechnology), N-Cadh (1:500; BD Transduction Laboratories), p75NTR (1:1000; Millipore), GAP43 (1:500; Millipore), c-Jun (1:1000; Cell Signaling Technology), and GAPDH (1:5000; Sigma-Aldrich). HRP-conjugated secondary antibodies (1:2000 in blocking solution) were from Cell Signaling Technology. For immunohistochemistry, incubation with MBP antibodies (1:10,000; Covance) or 324 rat anti-mouse Ig L1 antibodies (1:10) were followed by anti-mouse Ig AlexaFluor-488 (1:500; Invitrogen) or anti-rat Ig AlexaFluor-488 (1:500; Invitrogen), respectively. Incubation with antibodies to Ki67 (1:100; Abcam) and SOX10 (1:100; R&D Systems) were followed by biotinylated anti-rabbit IgG (1:600; GE Healthcare) and anti-goat Ig AlexaFluor-488 (1:1000; Invitrogen) antibodies, respectively. The Ki67 sections were then incubated with AlexaFluor-488 streptavidin (1:500; Invitrogen). Caspase-3 antibody (1:100; Cell Signaling Technology) was followed by anti-rabbit Ig Cy3. S100 antibody (1:1000) was from Dako.

##### Nerve injury.

The right sciatic nerve was exposed and transected at the sciatic notch (Woodhoo et al., 2009) or crushed (3 × 15 s at three rotation angles) using fine forceps. Contralateral uninjured sciatic nerves were used as controls.

##### Cell and segment cultures, BrdU assay, infection, and transfection.

Schwann cell cultures and BrdU assay were as in Morgan et al. (1991) (see also Survival assays). Mouse Schwann cells and Schwann cell precursors were prepaired as in Dong et al. (1999), Arthur-Farraj et al. (2012) and Jessen et al. (1994), respectively. The precursors were cultured in serum-free supplemented medium (Meier et al., 1999), referred to as defined medium, containing 20 ng/ml βNRG-1. Tibial nerve segments were maintained in DMEM with 5% FBS (Gomez-Sanchez et al., 2015). Adenoviral infections and plasmid transfections were as in Parkinson et al. (2001, 2004). An adenovirus expressing Cre recombinase (Akagi et al., 1997) was used to infect STAT3^f/f^ Schwann cells generating STAT3 KO cells. Constitutively active STAT3 plasmid (Bromberg et al., 1999), STAT3-CA, was provided by Dr. A. Stephanou (Institute of Child Health, University College London, London). The control used was the pRc/CMV empty vector (Invitrogen). Both were cotransfected with a pBabe-GFP plasmid to allow visualization of transfected cells. The STAT3 peptide inhibitor (Calbiochem) used in the proliferation assay is a cell-permeable STAT3-SH2 domain-binding phosphopeptide that contains a C-terminal membrane translocating sequence, acting as a highly selective, potent blocker of STAT3 activation (Turkson et al., 2001). The AG490 JAK2 kinase (STAT3) inhibitor was from Calbiochem.

##### Electron microscopy.

Nerves were processed as previously described (Gomez-Sanchez et al., 2015). Transverse ultrathin sections of cut tibial nerves were taken 5 mm from the cut site. To analyze the structure of bands of Büngner, 20–26 random photographs per nerve at X12K were used. For cell counts, nuclei counted in every field, or every second or every third field, depending on the size of the nerve, were multiplied by the number of fields to generate totals. Regeneration tracks (bands of Büngner) were identified as a group of Schwann cell profiles (sometimes a single profile) surrounded by a basal lamina sheath as seen in transverse nerve sections. Roundness index and profile area were obtained after manual tracing of randomly selected profiles using ImageJ software.

##### Survival assay.

Schwann cells from P1 STAT3 cKO and WT mice were assayed as in Meier et al. (1999). Cells were plated at low density (200 cells/coverslip) or high density (3000 cells/coverslip. After 3 h at 37°C and 5% CO_2_, one set of coverslips from each animal was fixed immediately for immunolabeling to obtain a reference point for the quantification of survival at later time points. The remaining sets were topped up with 400 μl of defined simple medium (sDM) (Meier et al., 1999) alone or sDM containing 1.6 ng/ml IGF-II (Peprotech), 0.8 ng/ml PDGF-BB (Peprotech), and 0.8 ng/ml NT-3 (Regeneron Pharmaceuticals), or conditioned medium, and cultured for 48 or 72 h. Then, cells were fixed using 4% PFA for 10 min, labeled with S100 antibodies and Hoechst dye, and the number of surviving Schwann cells counted. Survival percentage is the number of living cells present at 48 and 72 h as a percentage of the number of cells that had attached to the substrate in sister cultures at 3 h. sDM consists of 1:1 DMEM and Ham's F-12 supplemented with BSA (350 μg/ml). Schwann cell conditioned medium was prepared as previously described (Meier et al., 1999).

##### TUNEL staining.

To detect apoptotic cells, DNA fragmentation was labeled using the TUNEL method, using TUNEL enzyme (Roche) and TUNEL Lab Mix (Roche), according to the manufacturer's protocol. To identify TUNEL-positive nuclei from Schwann cells and macrophages, immunolabeling with S100 and F4/80 (1:100; AbD Serotec), respectively, was performed subsequently. Nuclei were stained using Hoechst dye.

##### Western blotting and qPCR.

For blotting, homogenates were obtained from injured and uninjured nerves as well as cultured nerve segments essentially as previously described (Gomez-Sanchez et al., 2015). Experiments were repeated at least three times with fresh samples, and representative pictures are shown. Densitometric quantification was by Image Lab 4.1 (Bio-Rad Laboratories). Measurements were normalized to loading control GAPDH. For PCR, total RNA was isolated using the RNeasy Lipid Tissue Mini Kit (QIAGEN) with a DNase I step performed to eliminate traces of genomic DNA. Real-time PCR was performed using CFX96 Real-Time PCR Detection System (Bio-Rad). PrecisionPLUS qPCR Mastermix with SYBR Green (Primerdesign) was used to detect double-stranded DNA. Primer sequences are described in Table 1.

##### Behavioral tests.

Experiments conformed to United Kingdom Home Office guidelines. Six mice per genotype were tested. Mice were tested before surgery to ensure that there were no differences in normal responses between the genetic backgrounds. Tests were performed as in Arthur-Farraj et al. (2012).

##### Statistical analysis.

Results are expressed as mean ± SEM. Statistical significance was estimated by Student's *t* test, one-way ANOVA, two-way ANOVA, or Mann–Whitney *U* test. A *p* value <0.05 was considered as statistically significant. Statistical analysis was performed using GraphPad software (version 6.0).

## Results

### STAT3 activation is seen in embryonic nerves and persists in adult Schwann cells

**Figure 1.**
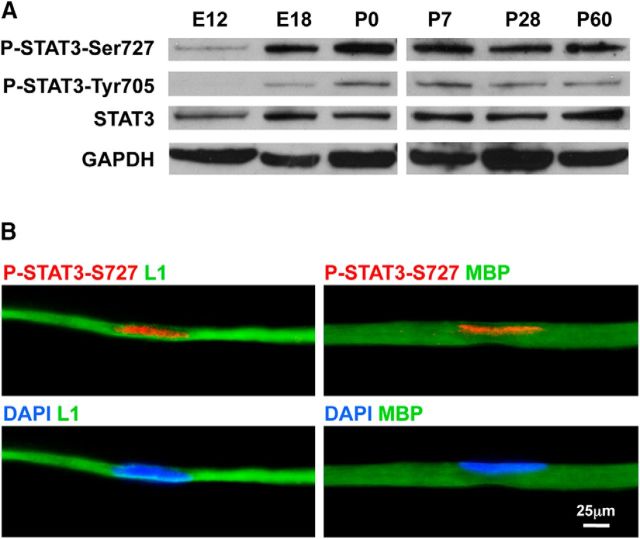
Basal activation of STAT3 takes place early in Schwann cell development and persists in the adult. ***A***, Western blots showing total STAT3 and its phosphorylated forms in sciatic nerve extracts from WT control mice at different developmental stages. STAT3 is present throughout the Schwann cell lineage. P-STAT3-Ser727 is found at very low levels in Schwann cell precursors (E12), but its expression increases at the immature Schwann cell stage and is maintained in adulthood. P-STAT3-Tyr705 is expressed at lower levels and seen from the immature Schwann cell stage (E18) onwards. GAPDH is used as a loading control. ***B***, Fibers in teased sciatic nerve preparations showing P-STAT3-Ser727 immunoreactivity in Schwann cell nuclei (red; top two panels) also stained with DAPI (bottom two panels). Two left panels, Nonmyelinated (Remak) fiber, identified by L1 antibodies. Right panels, Myelinated fiber, identified by MBP antibodies. Scale bar, 25 μm.

Before studying the role of STAT3 in Schwann cells, we analyzed STAT3 expression and activation during nerve development using Western blotting (Fig. 1*A*). STAT3 protein was present at all stages of the Schwann cell lineage from the Schwann cell precursor stage at embryo day 12 (E12) onwards. At the precursor stage, serine 727 and tyrosine 705 STAT3 phosphorylation (P-STAT3-Ser727 and P-STAT3-Tyr705) were low and undetectable, respectively. At the immature Schwann cell stage (E18), both P-STAT3-Ser727 and P-STAT3-Tyr705 were clearly upregulated and maintained until adulthood (Fig. 1*A*). Immunolabeling of teased adult nerves showed P-STAT3-Ser727 in the nucleus of both MBP-positive myelin cells and L1-positive nonmyelin (Remak) cells (Fig. 1*B*), although the lower levels of P-STAT3-Tyr705 could not be detected unambiguously by this method. Thus, STAT3 activation largely coincides with the Schwann cell precursor to Schwann cell transition, and basal STAT3 activation persists in adult nerves.

### In WT mice, STAT3 does not have a major role in Schwann cell development and myelination

**Figure 2.**
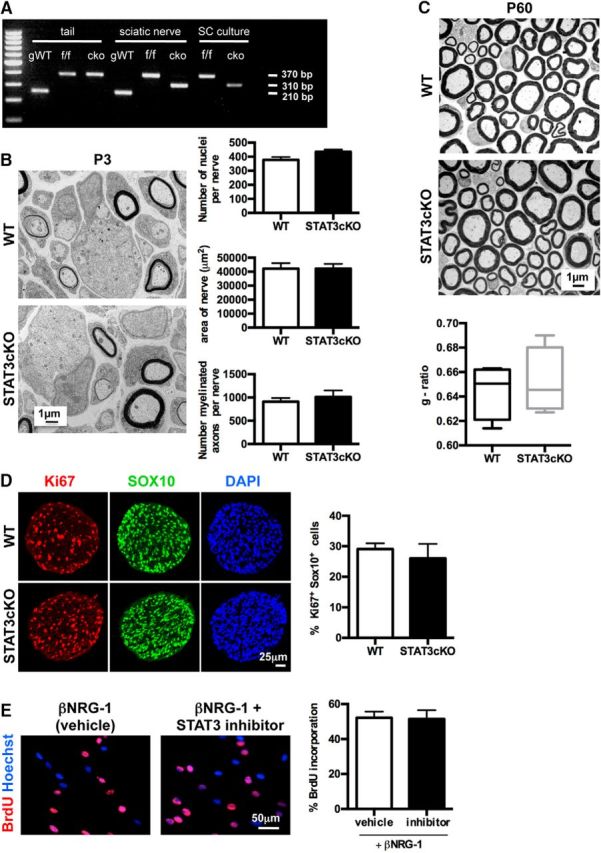
STAT3 appears dispensable for Schwann cell development and myelination. ***A***, Cre-mediated deletion of the STAT3 gene in Schwann cells. PCR analysis for STAT3 gene deletion in DNA extracts from tail and sciatic nerve of adult genetically wild-type (gWT), STAT3^f/f^ (f/f), and P_0_-Cre^+^STAT3^f/f^ (cKO) mice. Amplification of the gWT allele results in a 210 bp fragment, whereas STAT3^f/f^ allele containing the loxP sites generates a bigger band (370 bp). The deleted allele generates a 310 bp fragment. Specific recombination is absent from the tail of cKO mice but present in cKO sciatic nerve where Cre recombinase is expressed under the control of the P_0_ gene. Successful deletion in the STAT3 gene was also detected in DNA samples from P8 purified Schwann cell cultures from STAT3cKO mice. ***B***, Electron micrographs from P3 sciatic nerves of WT and STAT3cKO mice showing normal nerve morphology and no apparent abnormalities of myelination in the mutant. Graphs represent quantification of number of nuclei per transverse nerve section (top), nerve area (middle), and number of axons starting to be myelinated (bottom) in WT and STAT3cKO nerves. *n* = 5 mice of each genotype. Data are mean ± SEM. Scale bar, 1 μm. ***C***, Electron micrographs from adult sciatic nerves of WT and STAT3cKO mice showing normal myelin profiles in the mutant. Graph represents g-ratio analysis; the differences in g-ratios are not significant. *n* = 4 of each genotype. Data are mean ± SEM. Scale bar, 1 μm. ***D***, Ki67 immunolabeling showing similar Schwann cell proliferation in P1.5 sciatic nerves of WT and STAT3cKO mice. Transverse nerve sections were double-labeled for SOX10 and Ki67. Graph represents the percentage of Ki67^+^SOX10^+^ cells relative to total number of SOX10^+^ cells, showing no differences between groups. *n* = 4 for each genotype. Data are mean ± SEM. Scale bar, 25 μm. ***E***, BrdU immunolabeling showing that inhibition of STAT3 has no significant effect on Schwann cell proliferation induced by βNRG-1. Cultured, purified Schwann cells from WT mice were treated with βNRG-1 (20 ng/ml) for 48 h to stimulate proliferation with BrdU included for the last 24 h. The experiment was performed in the presence of vehicle or a STAT3 peptide inhibitor. Graph represents the percentage of BrdU^+^ nuclei relative to the number of Hoechst-stained nuclei. *n* = 4 for each genotype. Data are mean ± SEM. Scale bar, 50 μm.

To explore the potential importance of STAT3 in the Schwann cell lineage, we generated a conditional knock-out mouse in which STAT3 gene is specifically ablated only in Schwann cells. To do this, STAT3^f/f^ mice, having loxP sites flanking exons 12–14 of the STAT3 gene (Alonzi et al., 2001), were crossed with mice expressing Cre under the control of the P_0_ promoter (Feltri et al., 2002), to generate P_0_-Cre^+^/STAT3^f/f^ (STAT3cKO) mice (Fig. 2*A*).

The STAT3cKO mice were born and survived normally, and their nerves were indistinguishable from control^f/f^ littermates (WT). At postnatal day 3 (P3), the area of a transverse section through the sciatic nerve, the number of Schwann cell nuclei/nerve, and the number of myelinated axons/nerve were similar in STAT3cKO and WT mice (Fig. 2*B*). In adult nerves, thick myelin sheaths were seen around the largest caliber axons, and no significant difference was observed in the g-ratio between STAT3cKO and WT nerves (Fig. 2*C*).

The fact that Schwann cell numbers in the mutants were normal at P3 suggested that STAT3 signaling affected neither normal developmental death nor proliferation. This was confirmed by double-labeling sections of P1.5 sciatic nerves with the proliferation marker Ki67 and SOX10 antibodies to identify Schwann cells. No significant difference was found in the number of Schwann cells labeled with Ki67 antibodies between WT and STAT3cKO nerves (Fig. 2*D*). In another test of proliferation, purified cultures of mouse Schwann cells were treated with βNRG-1, a well-established mitogen for Schwann cells *in vitro*, in the presence of a STAT3 peptide inhibitor. BrdU incorporation revealed that STAT3 inhibition had no effect on DNA synthesis of Schwann cells (Fig. 2*E*). The same results were also seen using AG490, an inhibitor of the JAK2 signaling pathway (Nielsen et al., 1997) (data not shown).

Together, these results suggest that the basal STAT3 activation in embryonic and postnatal Schwann cells is largely dispensable and has little developmental significance. In lens development, functional redundancy between STAT3 and STAT1 has been suggested (Ebong et al., 2004), but this issue remains unclear (Hirahara et al., 2015). Nevertheless, it remains possible that, in a mouse in which STAT1 was genetically inactivated, a function for STAT3 in Schwann cell development might be revealed.

### In injured nerves, STAT3 is activated to support Schwann cell survival

**Figure 3.**
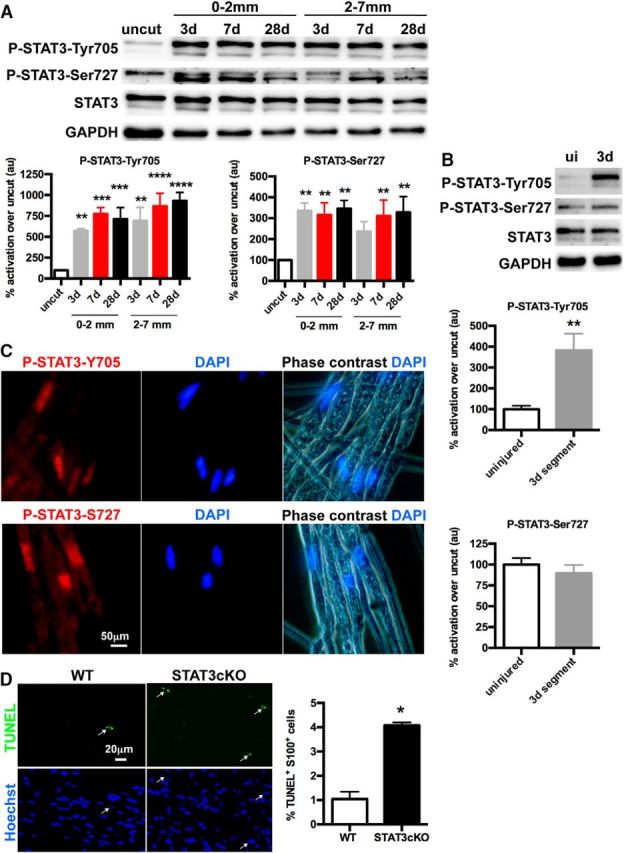
STAT3 activation after injury promotes Schwann cell survival. ***A***, Western blots showing the upregulation of P-STAT3-Tyr705 and P-STAT3-Ser727 distal to injury in uncut, as well as in 3, 7, and 28 d cut sciatic nerves from WT mice. The measurements were performed on 2 and 5 mm segments measured from the cut site as indicated. Densitometric quantification of Western blots shows the percentage of activation in cut relative to uncut nerves. *n* = a minimum of 4 mice per time point. Data are mean ± SEM. **p < 0.01, cut versus uncut (one-way ANOVA). ***p < 0.001, cut versus uncut (one-way ANOVA). ****p < 0.0001, cut versus uncut (one-way ANOVA). ***B***, Western blots comparing WT tibial nerve segments maintained *in vitro* for 3 d, to uninjured WT nerves. Note activation of P-STAT3-Tyr705 in the segments while P-STAT3-Ser727 levels remain as in uninjured nerves. Graphs represent the percentage of activation in segments relative to uninjured nerves. *n* = 5. Data are mean ± SEM. **p < 0.01 (Mann–Whitney *U* test). ***C***, Teased fibers of 3 d cut nerves from WT control mice labeled with P-STAT3-Tyr705 and P-STAT3-Ser727 antibodies. Positive immunostaining is localized in Schwann cell nuclei. DAPI labels nuclei. Scale bar, 50 μm. ***D***, TUNEL labeling of sections from the distal stump of sciatic nerve 3 d after cut. Note higher percentage of apoptotic nuclei in STAT3cKO mice compared with WT controls. Arrows indicate TUNEL-positive nuclei. Graph represents percentage of TUNEL/S100-positive cells in the Hoechst/S100-positive Schwann cell population. *n* = 4 for each genotype. Data are mean ± SEM. *p < 0.05 (Mann–Whitney *U* test). Scale bar, 20 μm.

STAT3 signaling promotes survival in a number of cell types (e.g., Schweizer et al., 2002; Shen et al., 2004). To determine whether STAT3 supports Schwann cell survival in injured nerves, we examined STAT3 activation in cut sciatic nerves and compared Schwann cell survival in cut nerves of WT and STAT3cKO mice. Western blots of cut nerves of adult WT mice showed a sharp (∼8- to 10-fold) rise in P-STAT3-Tyr705, the phosphorylation epitope that controls STAT3 dimerization and activation (Aaronson and Horvath, 2002). This was seen in nerve segments 0–2 mm and 2–7 mm distal to the cut at several time points, 3, 7, and 28 d, after injury (Fig. 3*A*). This STAT3 activation was not due to invading macrophages because it was also seen in distal segments from cut nerves maintained *in vitro* for 3 d under conditions where macrophages are unable to invade (Fig. 3*B*). P-STAT3-Ser727, generally thought to modify signaling mediated by P-STAT3-Tyr705 (Decker and Kovarik, 2000), was also elevated after injury (Fig. 3*A*), and immunolabeling for both P-STAT3-Tyr705 and P-STAT3-Ser727 was seen in Schwann cell nuclei in teased injured nerves (Fig. 3*C*). In cultured nerve segments, however, Western blots failed to show P-STAT3-Ser727 upregulation (Fig. 3*B*). This suggests that the activation of this epitope in Schwann cells after injury requires additional signals, which are present *in vivo* but not in culture. Alternatively, it is possible that macrophages contribute significantly the signal measured in *in vivo* nerve homogenates (Girolami et al., 2010). In mice, nerve cut results in apoptotic Schwann cell death in the distal nerve stump. To test whether STAT3 supported the survival of Schwann cells after injury, we quantified dying cells in sections from sciatic nerves 3 d after cut using the TUNEL assay. This revealed a fourfold increase in the number of S100-positive TUNEL-labeled Schwann cells in STAT3cKO nerves compared with control nerves (Fig. 3*D*). Confirming this, an increase in the number of caspase-3-positive cells was also seen in cut STAT3cKO nerves (data not shown).

**Figure 4.**
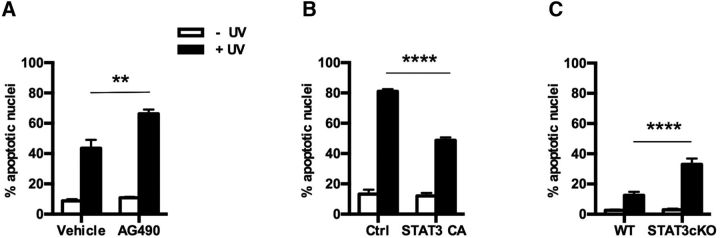
STAT3 protects Schwann cells from apoptosis after 24 h exposure to UV light. ***A***, Graph shows higher percentage of apoptotic nuclei in rat Schwann cells treated with AG490 (25 μm) to inhibit JAK2/STAT3 signaling compared with vehicle. *n* = 3 for each genotype. Data are mean ± SEM. **p < 0.01 (two-way ANOVA). ***B***, Graph shows lower percentage of cell apoptosis in rat Schwann cells expressing a constitutively active form of STAT3 (STAT3-CA). Cultured Schwann cells were cotransfected with STAT3-CA or an empty vector (pRcCMV) and GFP. *n* = 5 for each genotype. Data are mean ± SEM. ****p < 0.0001 (two-way ANOVA). ***C***, Graph showing percentage of apoptotic nuclei in STAT3^f/f^ mouse Schwann cells infected with a control GFP- (WT) or Cre-expressing (STAT3cKO) adenovirus. Cre-mediated deletion of the STAT3 gene in STAT3^f/f^ Schwann cells increased the percentage of UV-induced apoptosis. *n* = 5 for each genotype. Data are mean ± SEM. ****p < 0.0001 (two-way ANOVA).

To further test the idea that STAT3 is involved in the mechanisms that protect Schwann cells from death, we tested whether STAT3 protected cultured Schwann cells from stress induced by UV light, a model used to study STAT3 involvement in survival of other cell types (Shen et al., 2001; Sano et al., 2005). First, a UV time-course experiment determined that 24 h was optimal for assessing cell death (data not shown). At this point, Schwann cell nuclei started to show the hallmarks of apoptosis (condensed, bright nuclei fragmenting into apoptotic bodies), but the cells were still attached to the coverslip allowing quantification by Hoechst staining. Using rat Schwann cells, we found that inhibition of STAT3 signaling by the JAK2 inhibitor AG490 increased UV apoptosis (Fig. 4*A*). Conversely, enforced expression of a constitutively active form of STAT3 (STAT3-CA) protected Schwann cells from UV-induced death (Fig. 4*B*). Further, in mouse Schwann cells, UV light was more than twice as effective in inducing Schwann cell death in cells in which STAT3 had been genetically inactivated, compared with WT cells (Fig. 4*C*).

We conclude that activation of STAT3 signaling helps to protect Schwann cells in injured nerves from death.

### STAT3 is required for long-term survival of repair Schwann cells after injury

Injury-related Schwann cell death has chiefly been studied in two situations. One is the acute death examined above, which represents a transient phase of a strong increase in apoptosis from a very low level, in which a relatively small percentage of Schwann cell die (Grinspan et al., 1996; Yang et al., 2008; Ahmad et al., 2015). The other is the slow, large-scale death of Schwann cells that are deprived of axonal contact for long periods, often months, while axons regenerate toward them along the more proximal parts of the nerves. The loss of these chronically denervated Schwann cells is a major barrier to nerve repair in humans and has been extensively studied in rodents (Höke, 2006; Sulaiman and Gordon, 2009).

**Figure 5.**
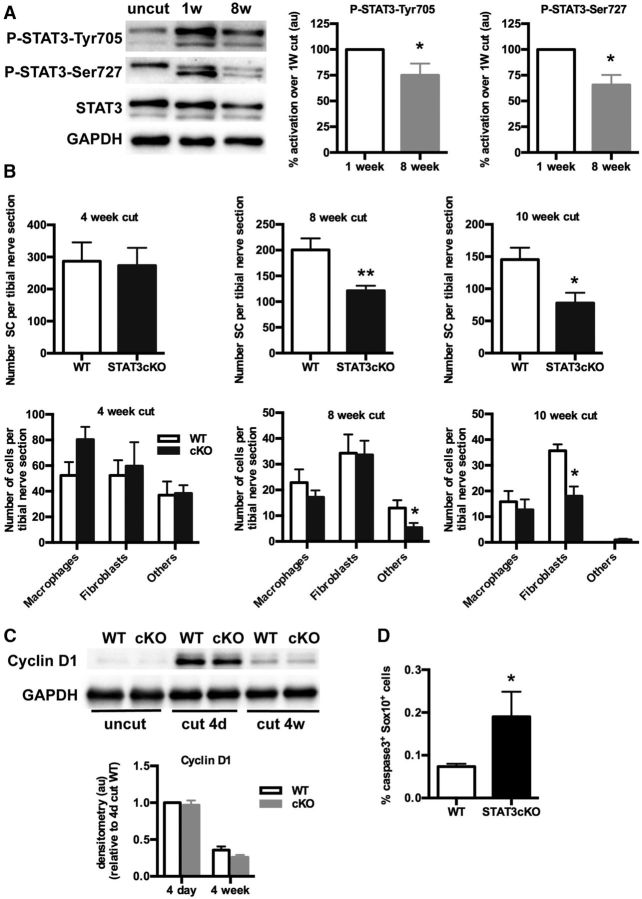
STAT3 promotes long-term survival of Schwann cells after nerve injury. ***A***, Western blots of uncut, and of 1 and 8 week cut nerves in WT mice showing that P-STAT3-Tyr705 and P-STAT3-Ser727 remain elevated at 8 weeks, although the levels are somewhat lower than at 1 week. Densitometric quantification shows the percentage of activation in 8 week cut nerves relative to 1 week cut nerves. *n* = 3 for each genotype. Data are mean ± SEM. *p < 0.05 (Wilcoxon's signed rank test). ***B***, Graphs representing the number of cell nuclei per transverse section of the tibial nerve. Electron micrographs of WT and STAT3cKO tibial nerves 5 mm distal to injury were used to identify and count cell types at 4 weeks (*n* = 3), 8 weeks (*n* = 7), and 10 weeks (*n* = 6) after sciatic nerve transection. Upper panels, Reduced number of Schwann cell nuclei in STAT3cKO tibial nerves 8 and 10 weeks after injury. Lower panels, No differences in macrophage number, but 10 week cut nerves from STAT3cKO mice had reduced numbers of fibroblasts. Data are mean ± SEM. *p < 0.05, STAT3cKO versus WT (Mann–Whitney *U* test). **p < 0.01, STAT3cKO versus WT (Mann–Whitney *U* test). ***C***, Western blots showing no differences in cyclin D1 expression in 4 d and 4 week STAT3cKO cut nerves compared with WT nerves. GAPDH is used as a loading control. Graph represents the densitometric analysis of the Western blot. Each value is normalized to that seen in 4 d cut WT nerves. *n* = 4 for each condition. Data are mean ± SEM. ***D***, Graphs show higher caspase-3 expression in 8 week cut nerves from STAT3cKO mice than in WT mice. *n* = 4 for WT and *n* = 3 for STAT3cKO. Data are mean ± SEM. *p < 0.05 (Mann–Whitney *U* test).

Having found that STAT3 protects against acute death, we tested whether STAT3 also regulated the loss of chronically denervated Schwann cells. In these experiments, we compared 1, 4, 8, and 10 week cut nerves in mice in which the proximal stump was deflected to prevent regeneration into the distal nerve stump. First, Western blotting showed that, in 8 week cut nerves, levels of P-STAT3-Tyr705 were only reduced by ∼20%–30% compared with those seen in 1 week cut nerves, in which P-STAT3-Tyr705 expression, in turn, is ∼8- to 10-fold that in uninjured nerves (see In injured nerves, STAT3 is activated to support Schwann cell survival). STAT3-Ser727 also remained activated in 8 week cut nerves (Fig. 5*A*). This shows that the STAT3 pathway remains activated in chronically denervated Schwann cells, a precondition for the involvement of STAT3 in maintaining this cell population. Second, to test whether this was the case, electron microscopy was used to count the number of Schwann cell nuclei in the distal stumps of transected tibial nerves of WT and STAT3cKO mice at 4, 8, and 10 weeks after cut. This showed that, in STAT3cKO nerves, the number of Schwann cells was substantially and significantly reduced compared with WT nerves at 8 and 10 weeks (Fig. 5*B*). This result matched with the higher number of caspase-3-positive Schwann cells found in 8 week cut nerves from STAT3cKO mice (Fig. 5*D*). At the earlier time point of 4 weeks, however, Schwann cell numbers were similar in both genotypes. The number of macrophages and fibroblasts was not significantly different in WT and mutant nerves at 4 and 8 weeks after cut, while the number of fibroblasts was reduced in the mutant nerves at 10 weeks (Fig. 5*B*). Light microscopic counts of cells in the nerves of the fourth toe of WT and STAT3cKO mice 4 weeks and 8 weeks after nerve cut (without reinnerveation) showed similar reduction in cell numbers to that found in more proximal nerves (above and data not shown).

Our previous data on the regulation of Schwann cell proliferation had suggested that STAT3 signaling was not involved (see In WT mice, STAT3 does not have a major role in Schwann cell development and myelination). This was confirmed by examining 4 d and 4 week cut nerves using cyclin D1 levels as a measure of proliferation (Atanasoski et al., 2001). At 4 d after cut, when proliferation is high, there was an equal and substantial rise in cyclin D1 levels in both WT and STAT3cKO nerves. Four weeks after cut, when proliferation is returning to baseline levels (Siironen et al., 1994; Hall, 1999), cyclin D1 levels were much lower and not significantly different between WT and mutants (Fig. 5*C*).

### STAT3 is required for autocrine survival signaling in denervated Schwann cells

**Figure 6.**
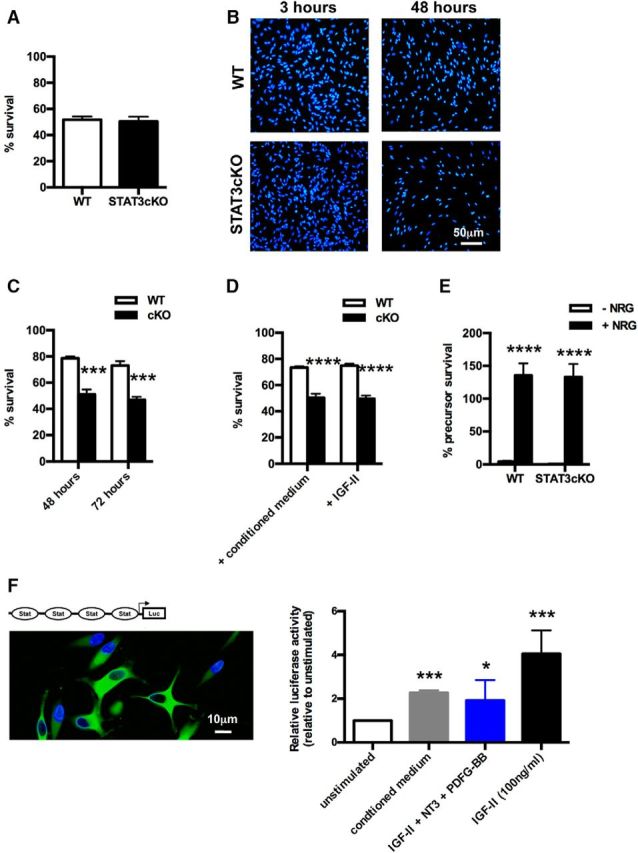
STAT3 is required for normal autocrine survival signaling by denervated Schwann cells. ***A***, Without autocrine support (at low density, 200 cells/400 μl of medium), STAT3cKO and WT Schwann cells show similar 48 h survival in sDM without serum. Survival is expressed as a percentage of cells present on sister coverslips 3 h after plating. *n* = 3. Data are mean ± SEM. ***B***, Hoechst staining shows nuclei of Schwann cells from P1 WT and STAT3cKO nerves cultured at high density (4000 cells/400 μl of medium) in sDM without serum for 3 and 48 h. There are reduced cell numbers in STAT3cKO cultures at 48 h. Scale bar, 50 μm. ***C***, With autocrine support (at high density, 4000 cells/400 μl), STAT3cKO Schwann cells survive poorly at 48 and 72 h compared with WT cells. The cells were cultured in sDM without serum. Survival is expressed as in ***A***. *n* = 3. Data are mean ± SEM. ***p < 0.001, STAT3cKO versus WT (two-way ANOVA). ***D***, Graph showing that low-density WT cultures respond to conditioned medium, or IGF-II at high concentration (100 ng/ml) by increase in survival at 48 h (compare with ***A***). STAT3cKO cultures do not show this response. *n* = 3. Data are mean ± SEM. ****p < 0.0001, WT versus STAT3cKO (two-way ANOVA). ***E***, WT and STAT3cKO Schwann cell precursors are equally responsive to the survival signal βNRG-1. Schwann cell precursors were dissected from STAT3cKO and WT E13 mouse embryos and cultured for 24 h with or without βNRG-1 (20 ng/ml). Survival was assessed as in ***A***. *n* = 4. Data are mean ± SEM. ****p < 0.0001, βNRG-1-treated versus untreated (two-way ANOVA). ***F***, Micrograph represents cultured rat Schwann cells infected with the STAT3-Luc-GFP adenovirus (green) and Hoechst nuclear staining (blue). Graph represents luciferase fluorescence, indicative of STAT3 activation, in response to 48 h exposure to conditioned medium, the combination of IGF-II (1.6 ng/ml), NT3 (0.8 ng/ml), and PDGF-BB (0.8 ng/ml) that mimics the conditioned medium, or high concentration of IGF-II (100 ng/ml). The activation is expressed relative to the signal obtained from control medium. *n* = 6 for conditioned medium and *n* = 12 for the combination of IGF-II, NT3, and PDGF-BB and high concentration of IGF-II. Data are mean ± SEM. *p < 0.05 (Kruskal–Wallis test). ***p < 0.001 (Kruskal–Wallis test). Scale bar, 10 μm.

The experiments above indicated that STAT3 signaling supports the short- and long-term survival of Schwann cells after injury, an issue of particular importance for regeneration. We therefore examined the underlying mechanism. Previously, we suggested that the survival of Schwann cells in injured nerves depended on autocrine signaling involving IGF, NT-3, and PDGF-BB (Meier et al., 1999). We also showed that during development autocrine circuits appear at the immature Schwann cell stage (E18) and are not present in Schwann cell precursors, a developmental timing that the present work shows coincides with STAT3 activation. To test whether STAT3 signaling plays a role in Schwann cell autocrine survival circuits, we performed experiments similar to those we used previously to identify autocrine Schwann cell mechanisms (Meier et al., 1999). First, to investigate the general ability of STAT3cKO cells to survive in culture without autocrine support (i.e., at low density), Schwann cells from P1 STATcKO and WT mice were plated at low density in sDM. In a 48 h assay, we found that the ability of STAT3cKO and WT cells to survive under these conditions was identical, both cells showing ∼50% survival relative to the cell number present 3 h after plating, which is in line with previous results (Meier et al., 1999) (Fig. 6*A*). When WT cells are plated at high density in this assay, their survival at 48 h increases to ∼80% due to autocrine factors secreted by the Schwann cells themselves (Meier et al., 1999). To test whether these autocrine survival circuits functioned without STAT3, cells from P1 STATcKO and WT mice were plated at high density. The survival of WT cells at 48 and 72 h increased to ∼80% as expected (Fig. 6*B*,*C*). The survival of STAT3cKO cells, however, remained similar to that seen in sparse cultures, suggesting absence of autocrine survival support (Fig. 6*B*,*C*).

In a further test of this, sparse cultures were exposed to conditioned defined medium previously conditioned by dense Schwann cell cultures. As expected from an autocrine mechanism, and shown in previous work, this increased the survival of sparsely plated WT cells to levels similar to those seen in densely plated cultures. In contrast, the survival of sparsely plated STAT3cKO cells was not increased in response to conditioned medium, or to IGF-II, the major constituent of the conditioned medium (Meier et al., 1999) (Fig. 6*D*). This confirmed the absence of functioning autocrine survival circuits in STAT3cKO cells and indicated that these cells are not responsive to autocrine signals, even when they are present in the culture medium. STAT3cKO cells remained, however, normally responsive to another key survival signal in the Schwann cell lineage, βNRG-1, which is expressed on axons and acts in a paracrine manner because βNRG-1 was equally effective in supporting the survival of E13 Schwann cell precursors from WT and STAT3cKO mice (Fig. 6*E*).

To determine whether autocrine Schwann cell signals activate endogenous STAT3 signaling, we used an adenoviral STAT3-luciferase GFP reporter construct containing four tandem copies of STAT3 binding sites (Besser et al., 1999, Staples et al., 2007) (Fig. 6*F*). The construct was infected into neonatal rat Schwann cell cultures before exposure to relevant components. Because low-density cultures generated insufficient luciferase signal, higher cell densities were used in these experiments, although this generated high background readings, even in control cultures without added factors, presumably due to the presence of autocrine factors. Nevertheless, elevated luciferase signal, indicating STAT3 activation, was obtained in response to Schwann cell conditioned medium, a combination of low concentrations of IGF-II, NT-3, and PDGF-BB that mimics the conditioned medium (Meier et al., 1999), or high concentration of the major conditioned medium ingredient IGF-II (Fig. 6*F*).

These results show that STAT3 is required for autocrine Schwann cell survival signaling, and suggest that defective autocrine survival support contributes to the substantial loss of STAT3cKO Schwann cells when these cells are subjected to chronic denervation.

### STAT3 is essential for the long-term maintenance of the phenotype of repair Schwann cells

The gradual loss of repair supportive capacity by distal nerves is due not only to the death of chronically denervated Schwann cells, but also to the gradual fading of the repair Schwann cell phenotype, evidenced by the gradual reduction in expression of regeneration supportive factors, such as GDNF and BDNF in distal nerve stumps during chronic denervation (Höke et al., 2002; Eggers et al., 2010). We therefore asked whether STAT3 might be more broadly involved in maintaining the regeneration-supportive functions of injured nerves by testing whether STAT3 was required for the maintenance of the repair Schwann cell phenotype, in addition to supporting the long-term survival of these cells. To this end, we compared the repair Schwann cell phenotype in 8 week cut distal stumps of WT and STAT3cKO nerves using morphometric analysis of regeneration tracks (bands of Büngner), qRT-PCR, and Western blotting.

**Figure 7.**
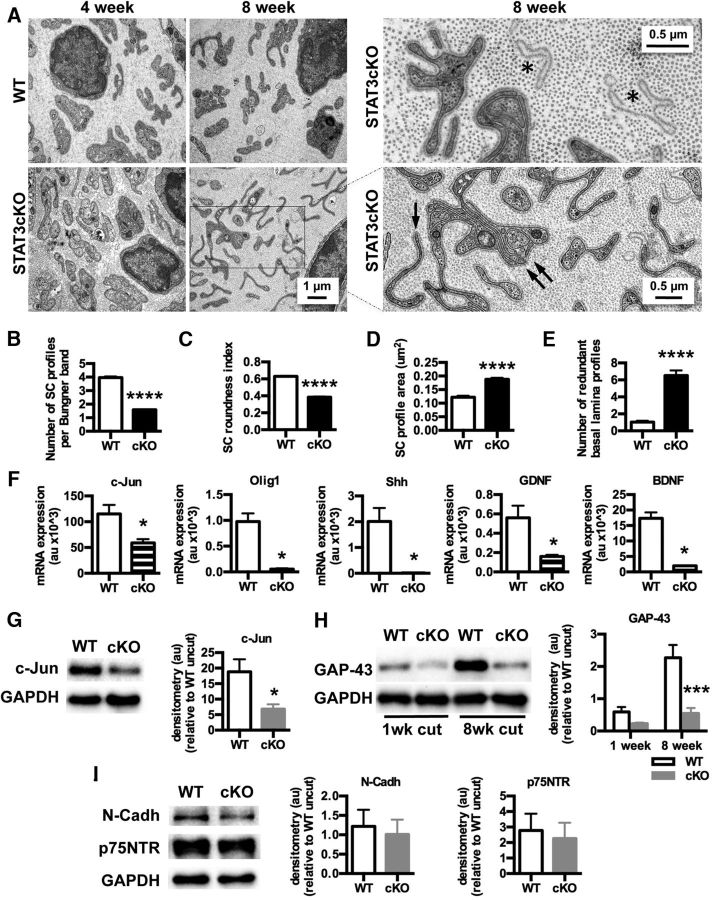
STAT3 is required for the maintenance of the repair Schwann cell phenotype. ***A***, Electron micrographs showing transverse sections of WT and STAT3cKO distal stumps at 4 and 8 weeks after transection (without regeneration). Note abnormal morphology of the regeneration tracks (bands of Büngner) in 8 week STAT3cKO nerves compared with 8 week WT controls. Right panels, Higher-power micrographs from STAT3cKO nerves illustrating redundant basal lamina (asterisks) and regeneration tracks containing a single (one arrow) or a few (double arrow) Schwann cell profiles, enclosed by a basal lamina. Scale bar, 1 μm. ***B–E***, Morphometric analysis of 8 week cut nerves. ***B***, The number of Schwann cell profiles per Büngner band is reduced by more than half in 8 week cut STAT3cKO nerves. *n* = 3. Data are mean ± SEM. ****p < 0.0001 (Mann–Whitney *U* test). ***C***, Schwann profiles in STAT3cKO regeneration tracks lose their roundness and become flatter. *n* = 3. Data are mean ± SEM. ****p < 0.0001 (Mann–Whitney *U* test). ***D***, The average area of Schwann cell profiles in STAT3cKO nerves is larger than in WT nerves. *n* = 3. Data are mean ± SEM. ****p < 0.0001 (Mann–Whitney *U* test). ***E***, STAT3cKO nerves show sixfold increase in the number of redundant basal laminae compared with WT samples. *n* = 3. Data are mean ± SEM. ****p < 0.0001 (Mann–Whitney *U* test). ***F***, qRT-PCR analysis showing significantly lower mRNA expression of the repair Schwann cell genes *c-Jun*, *Olig1*, *Shh*, *GDNF*, and *BDNF*, in 8 week cut distal nerves from STAT3cKO mice compared with WT controls. A pool of 9 WT and STAT3cKO distal stumps were used for RNA extraction. *n* = 3. Data are mean ± SEM. *p < 0.05 (Mann–Whitney *U* test). ***G***, Western blots showing lower expression of c-Jun in 8 week cut nerves from STAT3cKO mice compared with WT controls. *n* = 4 for each genotype. Data are mean ± SEM. *p < 0.05 (Mann–Whitney *U* test). ***H***, Western blots showing lower expression of GAP-43 in 8 week cut nerves from STAT3cKO mice compared with WT controls. Shown also are the relatively low GAP-43 levels 1 week after cut in both genotypes. Graphs represent the densitometric analysis of Western blots relative to WT uncut. *n* = 4 for each genotype. Data are mean ± SEM. ***p < 0.001 (two-way ANOVA). ***I***, Western blots showing similar levels of p75NTR and N-Cadherin in 8 week cut nerves of WT and STAT3cKO mice. *n* = 4 for each genotype. Graphs represent the densitometric analysis of Western blots relative to WT uncut. Data are mean ± SEM.

Morphologically, the regeneration tracks in STAT3cKO nerves were obviously abnormal. Compared with 8 week cut WT nerves, there were fewer cellular profiles in each track, the profiles were flatter, and the average area of each profile was increased. The number of redundant basal lamina profiles was also higher, a feature likely to reflect the increased cell death in these nerves (see previous section) (Fig. 7*A–E*).

Analysis of 8 week cut STAT3cKO nerves by qRT-PCR also showed substantial reduction in expression of key markers of repair Schwann cells, such as c-Jun, Olig1, and Shh, and repair-supportive factors, such as GDNF and BDNF (Fig. 7*F*), all of which are activated in Schwann cells after injury (Shy et al., 1996; Arthur-Farraj et al., 2012; Fontana et al., 2012; Brushart et al., 2013).

Western blotting showed that, 8 weeks after cut, STAT3cKO nerves expressed lower levels of c-Jun and GAP-43 proteins compared with WT (Fig. 7*G*,*H*). Two other proteins that are upregulated after injury, N-Cadherin and p75NTR, were expressed at levels similar to those seen in 8 week cut WT nerves (Fig. 7*I*).

**Figure 8.**
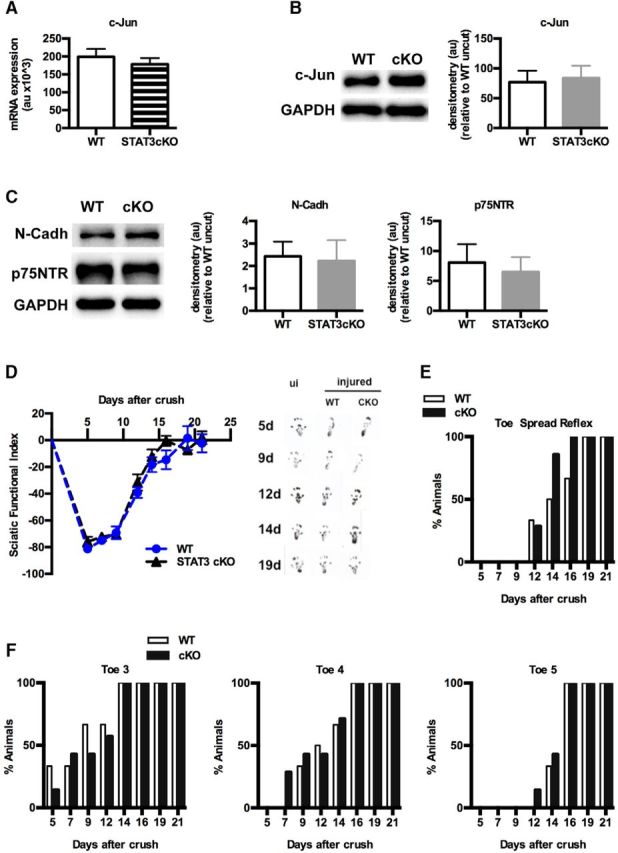
STAT3 is not required for the initial generation of the repair Schwann cell phenotype. ***A***, qRT-PCR analysis shows that, 1 week after cut, c-Jun expression in WT and STAT3cKO nerves is similar. RNA was extracted from 1 week cut nerves using a pool of 2 distal stumps from STAT3cKO and WT mice for each experiment. *n* = 4. Data are mean ± SEM. ***B***, Western blots showing similar c-Jun protein levels in 1 week cut WT and STAT3cKO nerves. Graphs represent the densitometric analysis of Western blots relative to WT uncut. *n* = 4 mice for each genotype. Data are mean ± SEM. ***C***, Western blots showing no differences in the expression of N-Cadherin and p75NTR proteins in WT and STAT3cKO nerves 1 week after cut. Graphs represent the densitometric analysis of Western blots relative to WT uncut. *n* = 4 mice for each genotype. Data are mean ± SEM. ***D***, Walking track analysis to quantify sensory-motor function following sciatic nerve injury. Sciatic function index (Inserra et al., 1998), which assesses nerve-mediated function of the hindlimb by measuring toe spread and print length of hindpaw footprints, showed similar values for WT and STAT3cKO mice before and, significantly, after nerve injury. Right, Representative examples of footprints from uninjured mice and at different times after injury as indicated. Note the increased print length and decreased toe spread after peripheral nerve lesion in both mouse lines. ui, Uninjured. *n* = 6. Data are mean ± SEM. ***E***, Toe spread reflex measurements show no significant differences in recovery of motor function between WT and STAT3cKO mice. *n* = 6. Data are mean ± SEM. ***F***, Toe pinch test for sensory function shows no significant differences between WT and STAT3cKO mice in the percentage of mice responding to pinching distal parts of toes 3, 4, and 5 after sciatic nerve lesion. *n* = 6. Data are mean ± SEM.

Whereas repair Schwann cells were clearly abnormal in 8 week cut STAT3cKO sciatic nerves, short-term denervated cells in these mice were relatively normal (Fig. 8). Thus, in 4 week cut nerves, the morphological changes that were obvious at 8 weeks were detectable but mild (Fig. 7*A*). In 1 week cut nerves, c-Jun mRNA and protein were found at normal levels in STAT3cKO mice. Expression of N-Cadherin and p75NTR protein was also similar in WT and STAT3cKO mice (Fig. 8*A–C*). In these nerves, the substantial difference in GAP-43 levels seen at 8 weeks was only emerging (Fig. 7*H*). In line with these findings, functional tests of regeneration of the sciatic nerve after crush injury indicated that nerve repair, which in these assays takes place during the first 2–3 weeks after injury, proceeds at a similar rate in WT and STAT3cKO mice (Fig. 8*D–F*).

We conclude that STAT3 has a dual role in injured nerves. It supports the long-term survival of repair Schwann cells and is required for the long-term maintenance of the repair Schwann cell phenotype. In contrast, STAT3 appears relatively unimportant for the initial generation of repair Schwann cells.

## Discussion

Because human nerves are long and axons grow slowly, all but the most distal nerve injuries result in chronic denervation of Schwann cells that can last for months, even years. In experimental animals, long-term denervation of the distal stump can be mimicked by nerve cut combined with deflection of the proximal stump to prevent reinnervation. Clinical observations and animal experiments agree that axon-free distal nerve stumps gradually lose the capacity to support regeneration. Although this is considered a key reason for regeneration failure in humans, and is known to involve loss of trophic factor expression and cell death, the molecular signaling mechanisms underlying this deterioration remain obscure (Höke, 2006; Sulaiman and Gordon, 2009; Jonsson et al., 2013). In the present work, we (1) report that STAT3 is activated by Tyr705 phosphorylation in Schwann cells distal to nerve injury in agreement with previous work by others, (2) show that this activation is sustained in repair Schwann cells during long-term denervation, and (3) demonstrate that selective inactivation of Schwann cell STAT3 results not only in a marked loss of Schwann cells from chronically denervated distal stumps, but also reduces the capacity of these cells to maintain their repair-supportive phenotype. This identification of a transcriptional mechanism involved in supporting long-term survival and differentiation of repair Schwann cells contributes to our understanding of how these important cells are maintained, and will help identify in molecular terms the failures that lead to their deterioration.

The present data indicate that STAT3 regulates Schwann cell survival, a function likely to be particularly significant for the long-term survival of Schwann cells in denervated distal stumps. This is in line with the role of STAT3 in other systems (e.g., Bader et al., 2014). We find that absence of STAT3 results in increased Schwann cell death in four different situations. First, this is seen in the STAT3cKO Schwann cells of chronically denervated distal nerves. Second, increased death is also seen STAT3cKO nerves 3 d after injury, although in this case relatively few Schwann cells die, and Schwann cell numbers are not significantly different between STAT3 mutants and WT controls (Grinspan et al., 1996; Yang et al., 2008; Ahmad et al., 2015). Third, STAT3 inactivation results in reduced response to autocrine survival signals in neonatal cultured Schwann cells. Fourth, STAT3 knock-out cells die more readily in response to UV irradiation.

STAT3 signaling does not appear to affect developmental death of Schwann cells, where we have previously shown that TGFβ signaling plays a role (D'Antonio et al., 2006), and it is not involved in Schwann cell precursor survival controlled by NRG-1. Although STAT3 signaling promotes proliferation in some systems (e.g., Debidda et al., 2005), we do not find this effect in Schwann cells. Together, our results suggest that the significant loss of repair Schwann cells in chronically denervated distal stumps of STAT3cKO mice is caused by a defective autocrine support, a survival mechanism that we and others have suggested to be central for preventing death of Schwann cells without axonal contact (Dowsing et al., 1999; Meier et al., 1999).

Using qRT-PCR analysis of the distal stump 8 weeks after nerve cut, we find that STAT3cKO mice show decreased expression of key markers of repair Schwann cells compared with levels seen in WT mice at the same time points. This includes *c-Jun*, *Olig1*, and *Shh* and of neurotrophic factors, such as *GDNF* and *BDNF* known to promote axonal regeneration. With respect to protein expression, GAP-43 and c-Jun levels were also lower, whereas N-Cadherin and p75NTR levels were the same in mutant and control distal stumps. This is significant because p75NTR promotes cell death in Schwann cells after nerve injury (Ferri and Bisby, 1999) but cannot be a defining factor in the increased death seen in STAT3 mutant distal stumps.

The importance of STAT3 signaling in the Schwann cells of long-term denervated nerves is also seen at the morphological level. In STAT3cKO mice, the shape of repair cells and structure of the regeneration tracks they form (bands of Büngner) is clearly altered, although not to the same extent as that seen in Schwann cell c-Jun null nerves (Parkinson et al., 2008; Arthur-Farraj et al., 2012). The number of Schwann cell profiles per Büngner band is also lower. This is likely to reflect the increased cell death in the mutants, but changes in cell shape could also contribute to this effect.

Elevation in STAT3 expression in injured nerves has been reported previously. Rapid activation of STAT3 signaling is implicated in the retrograde signaling from severed axons to the neuronal soma and in the initiation of axonal growth (Bareyre et al., 2011; Ben-Yaakov et al., 2012; Chandran et al., 2016). An increase in STAT3 signaling is also seen in Schwann cells after peripheral nerve injury. Sheu et al. (2000) used Western blotting to show activation of STAT3 Ser 727 in the proximal nerve stump from 30 min to 16 d after injury, in agreement with results above that show strong activation of STAT3 in axons after sciatic nerve transection. More modest activation was seen in the distal stump 3 and 24 h after nerve transection, most likely in Schwann cells. This finding was supported by Lee et al. (2009b) who showed activation of Ser727 in the sciatic nerve distal stump between 6 h and 5 d after crush injury and in cultured Schwann cells. In Schwann cells, IL-6, acting via gp130, and NRG-1, acting via ErbB2/3, can both induce STAT3 activation on Ser727 and Tyr705. IL-6, acting via STAT3, is also required for early induction of GFAP after nerve injury (Lee et al., 2009a, b).

We have shown previously, using immunohistochemistry, that GAP-43 levels in Schwann cells in the sciatic nerve rise slowly in the weeks after nerve injury (Curtis et al., 1992), although a rapid rise in GAP-43 levels is seen in terminal Schwann cells of the neuromuscular junction (Woolf et al., 1992). This is confirmed in the present study, which shows that in WT nerves protein levels of GAP-43 are higher at 8 weeks than at 1 week after nerve cut. In STAT3cKO nerves, however, levels of GAP-43 are substantially lower than those in WT, both at 1 week and, more strikingly, at 8 weeks after cut, suggesting that in Schwann cells STAT3 regulates GAP-43 levels. This is in line with that seen in other cell types, including neurons and astrocytes. In neurons, GAP-43 elevation after conditioning lesion depends on STAT3 and is promoted by the cytokine IL-6, which is also expressed at elevated levels in denervated Schwann cells (Hung et al., 2016). In astrocytes, STAT3 activation is involved in the activation of GAP-43 that occurs during astrogliosis (Cafferty et al., 2004; Qiu et al., 2005).

The prevention of the phenotypic deterioration and death of chronically denervated repair Schwann cells are two important goals in the effort to improve the outcomes after nerve injury. It is encouraging in this context that there is evidence, albeit limited, that the loss of the repair-supportive phenotype may be reversible. In rat Schwann cells, a reduction on the expression of c-erbB receptors and p75NTR due to long-term denervation can be restored *in vitro* by exposing the cells to NRG-1 (Li et al., 1998). When chronically denervated cells that have reduced growth-supportive capacity are treated *in vitro* with TGFβ, a factor expressed by macrophages and denervated Schwann cells, the regenerative support provided by these cells increases when they are tested in an *in vivo* grafting experiment (Sulaiman and Gordon, 2002). It has also been shown that engineered expression in Schwann cells of genes encoding particular growth associated factors can promote axon growth *in vivo* (Hoyng et al., 2014).

Although individual proteins will play a prominent role (Fontana et al., 2012), the exceptional capacity of repair Schwann cells to support regeneration is likely due to the integrated action of many components, including cell surface and secreted factors and morphology. The identification of pathways and signals, including transcription factors which determine this repair phenotype, opens the way toward pharmacological interventions that coordinately upregulate the repair program and therefore provide a favorable way of promoting nerve repair. c-Jun is one such signal because the striking elevation of Schwann cell c-Jun after injury acts as a global amplifier of the repair phenotype, without which regeneration is seriously curtailed (Arthur-Farraj et al., 2012). The question of whether c-Jun is also involved in the log-term maintenance of repair Schwann cells is under investigation. The present work identifies STAT3 as the second transcription factor that regulates the repair cell. Although STAT3 is less involved in the initial reprogramming of myelin and Remak cells to repair cells, it has a significant role in long-term denervated distal stumps, where STAT3 maintains the differentiation state of repair Schwann cells, and supports their survival. In future work, it will be of interest to explore this pathway and related signaling molecules for their potential to promote regeneration.
